# Levels of ACL-straining activities increased in the six months prior to non-contact ACL injury in a retrospective survey: evidence consistent with ACL fatigue failure

**DOI:** 10.3389/fphys.2023.1166980

**Published:** 2023-05-05

**Authors:** Louis H. Grodman, Mélanie L. Beaulieu, James A. Ashton-Miller, Edward M. Wojtys

**Affiliations:** ^1^ Medical School, University of Michigan, Ann Arbor, MI, United States; ^2^ Department of Orthopaedic Surgery, University of Michigan, Ann Arbor, MI, United States; ^3^ Department of Mechanical Engineering, University of Michigan, Ann Arbor, MI, United States; ^4^ Department of Biomedical Engineering, University of Michigan, Ann Arbor, MI, United States

**Keywords:** anterior cruciate ligament, injury, physical activity, fatigue, microdamage, sports

## Abstract

**Introduction:** Recent evidence has emerged suggesting that a non-contact anterior cruciate ligament (ACL) tear can result from repetitive submaximal loading of the ligament. In other words, when the intensity of ACL-straining athletic activities is increased too rapidly, microdamage can accumulate in the ligament beyond the rate at which it can be repaired, thereby leading to material fatigue in the ligament and its eventual failure. The objective of this survey-based exploratory study was to retrospectively determine whether the levels of various athletic activities performed by ACL-injured patients significantly changed during the 6 months before injury.

**Methods:** Forty-eight ACL-injured patients completed a survey to characterize their participation in various activities (weightlifting, sport-specific drills, running, jumping, cutting, pivoting/twisting, and decelerating) at three timepoints (1 week, 3 months, 6 months) prior to ACL injury. Activity scores, which summarized the frequency and intensity of each activity, were calculated for each patient at each time interval. A series of linear mixed-effects regression models was used to test whether there was a significant change in levels of the various activities in the 6-month period leading up to ACL injury.

**Results:** Patients who sustained a non-contact ACL injury markedly increased their sport-specific drills activity levels in the time leading up to injury (*p* = 0.098), while those patients who sustained a contact ACL injury exhibited no change in this activity during the same time period (*p* = 0.829). Levels of running, jumping, cutting, pivoting/twisting, and decelerating increased for non-contact ACL-injured patients but decreased for contact ACL-injured patients, though not significantly (*p* values > 0.10). Weightlifting activity significantly decreased leading up to injury among contact ACL-injured patients (*p* = 0.002).

**Discussion:** We conclude that levels of ACL-straining athletic activities or maneuvers in non-contact ACL-injured patients markedly increased in the 6 months leading up to their injury, providing evidence that changing levels of certain activities or maneuvers may play a role in ACL injury risk. This warrants further investigation of the hypothesis that too rapid an increase in activities or maneuvers known to place large loads on the ACL can cause microdamage to accumulate in the ligament, thereby leading to failure.

## 1 Introduction

Recently, the prevailing dogma that non-contact anterior cruciate ligament (ACL) injuries are the result of a single catastrophic maneuver that overloads the healthy ACL has been challenged. While this can happen on occasion, evidence is mounting for a second injury mechanism: namely, that of “tissue fatigue”, by which repetitive submaximal loading of the ACL can cause microdamage to accumulate in the ligament, thereby weakening it until it causes the ACL to fail (Lipps et al., 2013; Wojtys et al., 2016; Chen et al., 2019; Kim et al., 2022). For instance, in an *in vitro* simulation of repetitive pivot-landings known to place the ACL under marked strain, the ACLs of cadaveric knees failed after fewer than 100 of those submaximal loading cycles (Lipps et al., 2013). Additionally, ACL tissue microdamage found in another group of cadaveric knees subjected to these repetitive pivot-landings (Kim et al., 2022) proved to be similar to that found to have accumulated in *ex vivo* ACL collagen fibrils and fibers of non-contact ACL injury patients at the time of surgery (Chen et al., 2019). Therefore, the ACL may fail when the accumulation of microdamage outpaces repair (i.e., catabolic state), similar to ulnar collateral ligament (UCL) injuries in baseball pitchers. In these athletes, it has long been established that repetitive throwing at high velocities without proper recovery can lead to an UCL tear (Mirowitz and London, 1992). For this reason, the total volume and frequency of pitches are monitored and limited in baseball. With regard to the ACL, however, there is as yet no such *in vivo* evidence that it can fail via repetitive sub-maximal loading. The focus of this study, therefore, is to address this knowledge gap by testing our overall hypothesis that if the ACL can fail via a ‘tissue fatigue’ injury mechanism, we would expect the frequency and/or intensity of activities/maneuvers that significantly strain the ACL (hereafter referred to as “ACL-straining athletic activities or maneuvers”) to have increased substantially prior to a patient’s ACL non-contact injury, before the ACL has had adequate time to adapt. In such cases, natural repair homeostasis would be replaced by a catabolic state where the rate of ACL tissue degradation exceeds that of synthesis, leading to an eventual ligament failure.

The purpose of this exploratory study was to determine whether the frequency and/or intensity at which ACL-injured patients performed various athletic activities or maneuvers known to markedly strain the ACL changed in the 6-month period leading up to their ACL injury. We selected a 6-month period because we estimated that given the ACL’s relatively slow repair rate (Rucklidge et al., 1992), it would not be able to adapt to a substantial increase in ACL-straining athletic activities or maneuvers within this timeframe. Although the exact repair rate of the ACL is unknown, we do know that ligaments heal more slowly than other tissues (Panjabi, 2006; Jung et al., 2009) given that ligaments have lower metabolic activity rates than muscle, bone, or cartilage (Robi et al., 2013; Nyland et al., 2022). In the present study, ACL reconstruction patients completed a questionnaire to retrospectively quantify the frequency and intensity of their athletic activity/maneuver leading up to injury. The questionnaire was designed to collect data on various athletic activities and maneuvers, in particular those that are known to significantly strain the ACL and have been associated with ACL injuries. Through video analysis for instance, non-contact ACL injuries have been found to occur while cutting/changing direction, pivoting/twisting, decelerating, and landing from a jump (Olsen et al., 2004; Krosshaug et al., 2007; Boden et al., 2009; Walden et al., 2015; Della Villa et al., 2020). We hypothesized that the frequency/intensity of ACL-straining athletic activities or maneuvers (i.e., decelerating, jumping, cutting, pivoting/twisting, sport-specific drills) would markedly increase in the 6-month period leading up to ACL injury, especially among those patients that sustained a non-contact injury.

## 2 Materials and methods

This study was a retrospective case series survey study. Patients having suffered an ACL injury were asked to complete one questionnaire post-injury to quantify the frequency and intensity at which they performed various athletic activities/maneuvers in the 6-month period leading up to their injury. In particular, it retrospectively quantified these data for the 6-month, 3-month, and 1-week pre-injury timepoints.

### 2.1 Participants

We recruited a convenience sample of 48 ACL-injured patients (12 males/36 females; age: 19.1 ± 6.5 years; height^†^: 1.7 ± 0.1 m; body mass^†^: 67.3 ± 12.7 kg; BMI^†^: 23.6 ± 4.0 kg/m^2^; ^†^data based on 47 patients due to missing data) who voluntarily completed a questionnaire at their initial post-injury visit (on average^†^, 56.8 ± 118.7 days post-injury) to determine their activity patterns leading up to their ACL injury. Most of the patients were athletes (46/48, 96%; playing experience: 11.4 ± 7.7 years), with most athletes participating in sports deemed risky for ACL injury, such as soccer and basketball. Many participants were also multi-sport athletes (Table 1). All ACL injuries were confirmed by physician examination and magnetic resonance imaging (MRI) evaluation. Fifteen (6 male/9 female) patients suffered a contact ACL injury, meanwhile 33 (6 male/27 female) patients suffered a non-contact injury. We excluded patients with a previous injury to the ACL and/or meniscus of their ipsilateral knee. All qualifying patients of the senior author at MedSport, the sport medicine clinic of the University of Michigan who sustained a primary ACL injury from 2016 to 2019 were invited to participate in this study. This study (HUM00109196) was approved by the Institutional Review Board of the University of Michigan Medical School (IRB00001996).

**TABLE 1:** Frequency of Sports in which Patients Participated Prior to ACL Injury.

Sport	Frequency total # (M/F) % of total patients (% of M/F)
Multi-sport	18 (6/12) 37.5% (50.0/33.3)
Ball sports	
Soccer	17 (3/14) 35.4% (25.0/38.9)
Basketball	12 (5/7) 25.0% (41.7/19.4)
Volleyball	6 (0/6) 12.5% (0/16.7)
Lacrosse	7 (0/7) 14.6% (0/19.4)
Football	5 (5/0) 10.4% (41.7/0)
Softball	4 (0/4) 8.3% (0/11.1)
Baseball	1 (1/0) 2.1% (8.3/0)
Rugby	1 (0/1) 2.1% (0/2.8)
Golf	1 (0/1) 2.1% (0/2.8)
Combined discipline/running/cycling	
Track & field	4 (2/2) 8.3% (16.7/5.6)
Dance	3 (0/3) 6.3% (0/8.3)
Cross country/running	2 (0/2) 4.2% (0/5.6)
Biking	1 (1/0) 2.1% (8.3/0)
Snow Sports	
Skiing	3 (0/3) 6.3% (0/8.3)
Snowboarding	2 (1/1) 4.2% (8.3/2.8)
Combat sports	
Wrestling	2 (2/0) 4.2% (16.7/0)
Mixed martial arts/Brazilian jiu-jitsu	1 (0/1) 2.1% (0/2.8)
Water Sports	
Swimming	1 (0/1) 2.1% (0/2.8)
Water polo	1 (1/0) 2.1% (8.3/0)
Rowing	1 (0/1) 2.1% (0/2.8)
No Sport	2 (0/2) 4.2% (0/5.6)

M: male patients; F: female patients.

### 2.2 Sport and Physical Activity Questionnaire

Each participant completed a “Sport & Physical Activity Questionnaire” we developed, which retrospectively assessed the level of various athletic activities and maneuvers of the participant in the 6-month period prior to their ACL injury (see Supplemental Material file). We divided the questionnaire into three identical sections, each corresponding to a different time point prior to injury: 6 months, 3 months, and 1 week. Within each section, we asked patients questions about their activity patterns for seven different activities: weightlifting, sport-specific drills, running, jumping, cutting, pivoting/twisting, and decelerating. They described the nature of their participation relating to each activity by three measures: 1) indicating (Yes or No) whether they participated in the activity; 2) providing the frequency (minutes per day and days per week) at which they participated in the activity; and 3) rating the intensity at which they participated in the activity on a 0–10 scale (0 = Not intense; 10 = Most intense).

The athletic activities or maneuvers of interest in the questionnaire were not mutually exclusive. In other words, it was possible for a participant to simultaneously engage in several of the activities or maneuvers during a single sporting/exercise session. This is especially true for the many patients that participated in sports that utilize several of the activities of interest. For example, a patient that participated in two 90-min soccer practices and one 30-min running bout per week could have a total frequency of 210 min/week for the “running” activity and could have a total frequency of 180 min/week for the “sport-specific drills”, “jumping,” “cutting,” “pivoting/twisting,” and “decelerating” activities (i.e., soccer typically includes the activities of running, sport-specific drills, jumping, cutting, pivoting/twisting, and decelerating).

### 2.3 Quantitative analysis

**FIGURE 1:**
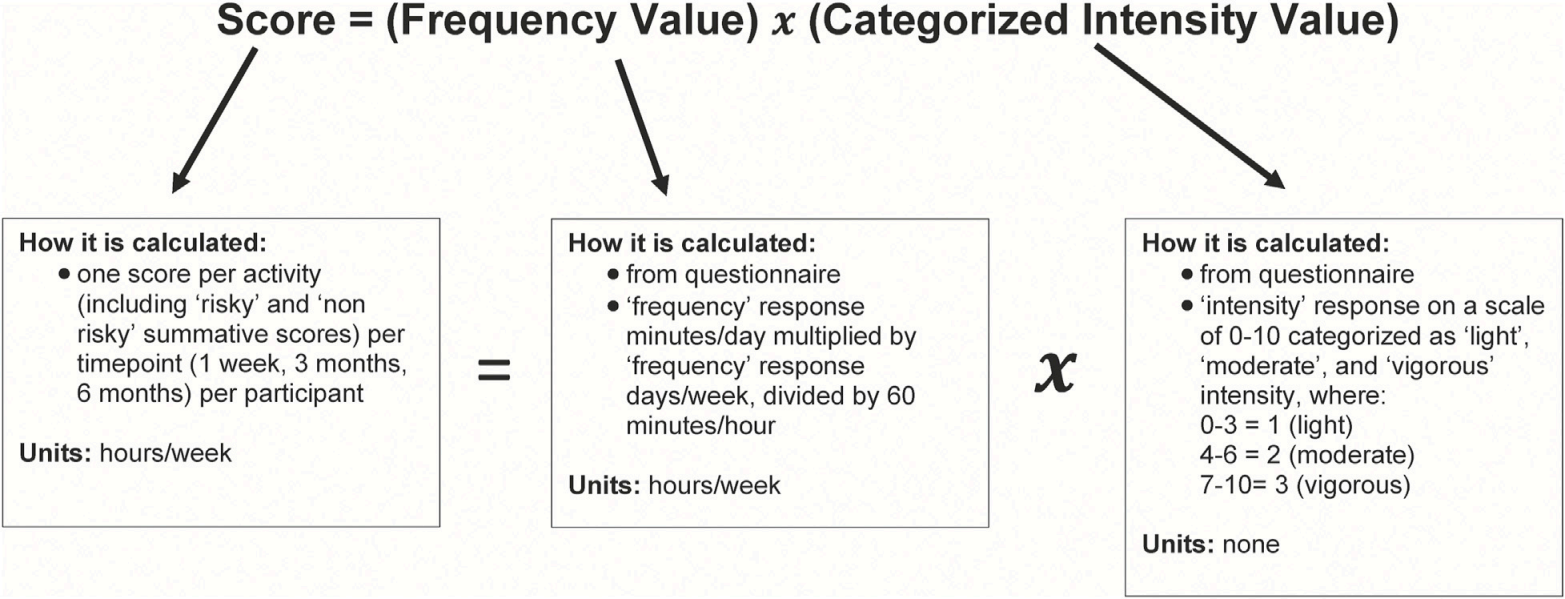
Activity score calculation. Diagram illustrating the calculation of activity scores. Diagram describes each component of the score calculation: the score, frequency value, and categorized intensity value. We retrieved the frequency value and categorized intensity value from patient questionnaires.

For analysis purposes, we categorized ACL-straining athletic activities or maneuvers (cutting, jumping, pivoting/twisting, decelerating, and sport-specific drills) as ‘*risky*’. Sports-specific drills were categorized as “risky” because nearly all the participants practiced a sport that included a considerable amount of cutting, jumping, pivoting/twisting, or decelerating in the sports’ basic maneuvers. We categorized activities or maneuvers not deemed risky for ACL injury (weightlifting and running) as “*non-risky*”. We summarized the frequency and intensity at which the participants partook in each activity or maneuver by a single score for each timepoint for each participant. We calculated this score using a custom formula that was created in consultation with an experienced statistician with our institution’s statistical consulting services. The formula multiplies the participants’ responses for frequency (calculated in ‘hours per week’) and a categorized intensity value based on their intensity response (Figure 1). The intensity values were categorized as ‘light’, ‘moderate’, and ‘vigorous’ intensity. The categorization was as follows: 1 for “light” intensity (patient response values 0–3); 2 for ‘moderate’ intensity (values 4–6); and 3 for “vigorous” intensity (values 7–10). The grouping of questionnaire intensity values was selected to parallel those of the Physical Activity Guidelines for Americans (Olson et al., 2018), which categorizes intensity values of 5–6 (on a 1–10 scale) as moderate intensity and 7–8 as the minimum for vigorous intensity. Additionally, we created summative “risky” and “non-risky” scores—the sum of the scores of all activities or maneuvers deemed “risky” and “non-risky”, respectively, in terms of ACL injury risk as listed above—for each participant at each timepoint.

### 2.4 Statistical analysis

We used a series of linear mixed-effects regression models to test whether there was a significant change in the levels of the aforementioned activities or maneuvers in the 6-month period leading up to ACL injury. The outcome variable for each model was the activity *score* for each activity or maneuver type (i.e., weightlifting, sport-specific drills, running, jumping, cutting, pivoting/twisting, decelerating, all “risky” activities or maneuvers, and all “non-risky” activities or maneuvers). The predictor variables for all models were *time*, *injury type*, the interaction of *time x injury type* and *patient*. The variables *time* and *injury type* were treated as fixed effects, where *time* was a continuous variable representing the number of days relative to injury (6 months = −182.5 days; 3 months = −91.25 days; 1 week = −7 days; injury = 0 days) and *injury type* was a categorical variable (coded as 0 = non-contact and 1 = contact ACL injury). We treated *patient* as a random effect. In addition, *post hoc* analyses were performed using data obtained from the regression models. First, we statistically compared the “time vs. score” slope of each injury type group to zero to test whether there was a significant change in levels of certain activities or maneuvers in the 6-month period leading up to ACL injury in each group. Second, we calculated the estimated marginal means of the activity scores for the three times points for each activity or maneuver type. These data were used for graphical purposes. Data were analyzed in R version 3.6.3 (R Core Team, 2020). The linear mixed-effects models were created with the lmer() function of the lme4 package (Bates et al., 2015). The statistical significance of the slopes were tested using the emtrends() functions of the emmeans package (Lenth et al., 2020). Lastly, the estimated marginal means were calculated with the emmeans() function also of the emmeans package. We set the alpha level for statistical significance at 0.10.

## 3 Results

In those patients that identified themselves as athletes, most ACL injuries occurred during their season (33/46; 72%), with fewer injuries occurring in the preseason (7/46;15%) and offseason (5/46;11%); one patient did not provide this information. Of all the patients (female/male patients), 66.7% (F: 61.1%/M: 83.3%), 89.1% (F: 88.2%/M: 91.7%), 91.1% (F: 88.2%/M: 100%), 86.4% (F: 87.9%/M: 81.8%), 76.6% (F: 77.1%/M: 75.0%), 95.6% (F: 94.1%/M: 100%), and 82.2% (F: 79.4%/M: 90.9%) participated in weightlifting, sport-specific drills, running, jumping, cutting, pivoting/twisting, and decelerating activities, respectively.

**FIGURE 2:**
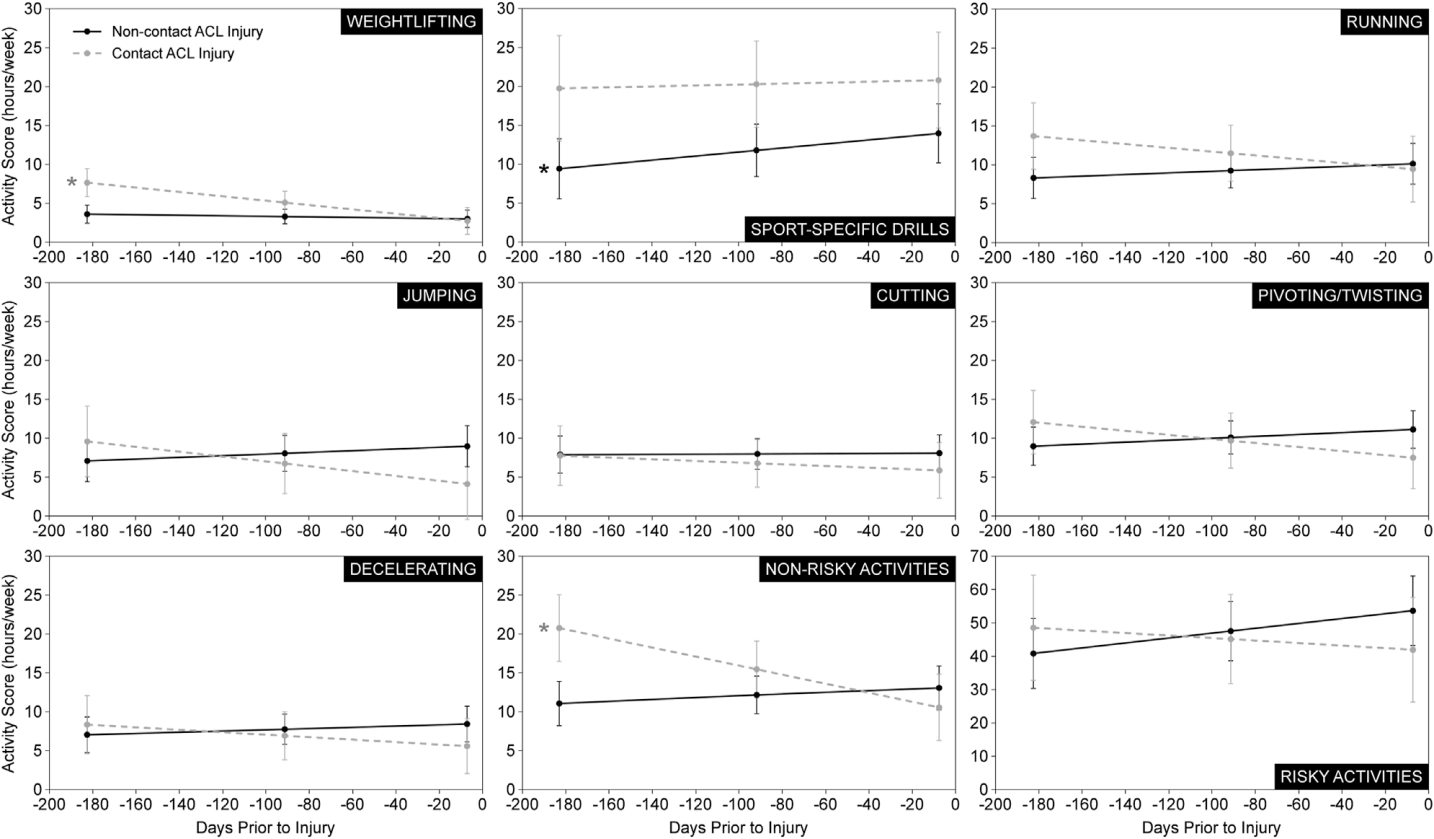
Estimated marginal mean activity scores. Estimated marginal mean activity scores (± 1 standard error) at each time point (6 months, 3 months, 7 days) prior to injury for each ACL injury group. Data are presented for all seven athletic activities and maneuvers in addition to the summative risky and non-risky activities. *Statistically significant slope (*p* < 0.10) indicating a significant change in athletic activity and maneuver levels leading up to ACL injury.

The statistics of the linear mixed-effects regression models for all athletic activities or maneuvers of all patients are presented in Table 2. A graphical representation of the change in each activity or maneuver’s score over the 6-month period leading up to ACL injury, for each injury group, can be found in Figure 2. Interestingly, patients who sustained a non-contact ACL injury significantly increased their sport-specific drills activity (i.e., score) in the 6-month period leading up to injury; meanwhile no change was observed in the sport-specific drills activity in the patients who sustained a contact injury (Table 2; Figure 2). In addition, the linear mixed-effects regression model for weightlifting revealed that patients engaged in significantly decreasing weightlifting activity over the 6-month period leading up to injury (Table 2; Figure 2). When breaking down the results by injury type, contact ACL injury patients significantly decreased their weightlifting activity leading up to injury; meanwhile no change was noted in weightlifting activity in the non-contact injury patients (Table 2; Figure 2). Similarly, contact ACL-injured patients also significantly decreased their combined non-risky activities or maneuvers, which includes weightlifting, leading up to injury (Table 2; Figure 2).

**TABLE 2:** Statistics of the Linear Mixed-Effects Regression Models.

Variable	EMS/β^*a*^	SE^*a*^	p value^*a*^	L_95%CI^*a*^	U_95%CI^*a*^
Weightlifting					
Time—all patients	−0.016	0.005	**0.003**	−0.026	−0.005
Time—noncontact injuries	−0.003	0.006	0.542	−0.015	0.008
Time—contact injuries	−0.028	0.009	**0.002**	−0.046	−0.011
Time*injury type	−0.025	0.010	**0.020**	−0.045	−0.004
Sport-specific drills					
Time—all patients	0.016	0.016	0.316	−0.016	0.048
Time—noncontact injuries	0.026	0.016	**0.098**	−0.005	0.057
Time—contact injuries	0.006	0.028	0.829	−0.049	0.061
Time*injury type	−0.020	0.032	0.529	−0.082	0.042
Running					
Time—all patients	−0.007	0.011	0.540	−0.029	0.016
Time—noncontact injuries	0.011	0.012	0.378	−0.013	0.034
Time—contact injuries	−0.024	0.019	0.208	−0.062	0.014
Time*injury type	−0.035	0.023	0.126	−0.079	0.009
Jumping					
Time—all patients	−0.010	0.011	0.375	−0.033	0.013
Time—noncontact injuries	0.011	0.011	0.347	−0.012	0.034
Time—contact injuries	−0.031	0.020	0.119	−0.070	0.008
Time*injury type	−0.042	0.023	**0.070**	−0.087	0.003
Cutting					
Time—all patients	−0.005	0.011	0.652	−0.026	0.017
Time—noncontact injuries	0.001	0.012	0.929	−0.022	0.024
Time—contact injuries	−0.011	0.018	0.552	−0.047	0.025
Time*injury type	−0.012	0.021	0.583	−0.054	0.030
Pivoting/Twisting					
Time—all patients	−0.007	0.010	0.478	−0.026	0.012
Time—noncontact injuries	0.012	0.010	0.237	−0.008	0.033
Time—contact injuries	−0.026	0.016	0.115	−0.059	0.007
Time*injury type	−0.038	0.019	**0.051**	−0.076	−0.000
Decelerating					
Time—all patients	−0.004	0.010	0.693	−0.024	0.016
Time—noncontact injuries	0.008	0.011	0.471	−0.014	0.029
Time—contact injuries	−0.016	0.017	0.349	−0.049	0.018
Time*injury type	−0.024	0.020	0.239	−0.063	0.015
Non-risky activities					
Time—all patients	−0.024	0.014	**0.093**	−0.051	0.004
Time—noncontact injuries	0.011	0.014	0.441	−0.017	0.040
Time—contact injuries	−0.058	0.024	**0.016**	−0.105	−0.011
Time*injury type	−0.069	0.028	**0.014**	−0.123	−0.014
Risky activities					
Time—all patients	0.017	0.063	0.784	−0.108	0.143
Time—noncontact injuries	0.073	0.061	0.236	−0.049	0.194
Time—contact injuries	−0.038	0.110	0.732	−0.258	0.182
Time*injury type	−0.111	0.125	0.381	−0.354	0.138

Abbreviations: EMS, estimated marginal slope; SE, standard error; L_95%CI: lower limit of 95% confidence interval; U_95%CI: upper limit of 95% confidence interval; units of “time” are days; bold values indicate statistical significance (*p* < 0.10).^*a*^ refers to the estimated marginal slope data for the “time” variables and to the regression coefficient (*β*) data for the “time*injury type” interaction.

The change in the frequency/intensity of the running, jumping, cutting, pivoting/twisting, and decelerating activities or maneuvers, as well as the summative risky and non-risky activities or maneuvers in the 6 months leading up to ACL injury exhibited an interesting trend. Levels tended to increase in the non-contact ACL injury participants but tended to decrease in the contact injury participants (Figure 2). In fact, the change leading up to injury in levels of weightlifting, jumping, pivoting/twisting, and the summative non-risky activities/maneuvers was significantly different between patients who sustained a contact ACL injury and those who sustained a non-contact ACL injury (Table 2; Figure 2).

## 4 Discussion

This retrospective study investigated whether the frequency and/or intensity at which ACL-injured patients performed various athletic activities or maneuvers significantly changed in the 6-month period leading up to their ACL injury. Our results indicate that indeed there was a change in the frequency/intensity of several activities or maneuvers in the months leading up to injury. There was a significant increase in the frequency/intensity of sport-specific drills in patients who sustained a non-contact ACL injury; and there was a significant decrease in the frequency/intensity of weightlifting and summative non-risky activities or maneuvers in those who sustained a contact ACL injury. In general, this study illustrated a trend for levels of athletic activities or maneuvers deemed “risky” in terms of ACL injury to increase leading up to a non-contact ACL injury and for levels of ‘non-risky’ activities/maneuvers to decrease leading up to a contact injury. The rate at which the levels of various athletic activities or maneuvers changed in the 6-month period leading up to injury was significantly different between non-contact and contact ACL injury patients. In particular, this rate of change was significantly different between injury mechanism groups for jumping, pivoting/twisting, and weightlifting.

The significant increase in the frequency and/or intensity of sport-specific drills activity leading up to a non-contact ACL injury is consistent with the notion that the ACL can fail due to the accumulation of microdamage when subjected to submaximal repetitive loading (i.e., ‘tissue fatigue’ injury). The general trend of increasing levels of athletic activities and maneuvers deemed ‘risky’ in terms of ACL injury risk amongst non-contact ACL injury patients prior to injury is also in line with this notion. This is because a marked increase in the intensity and/or frequency of ACL-straining athletic activities and maneuvers, without allowing enough time for the ACL to adapt, may cause the ligament to transition from a state of homeostasis where its repair rate matches its rate of microdamage to a catabolic state, leading to progressive weakening and then the eventual failure of the ACL under normal conditions. A good example of this tissue homeostasis, or lack thereof, in the sports world is the UCL injury in baseball pitchers. It has been established that the UCL can sustain a “tissue fatigue” injury whereby microdamage accumulates in the ligament of pitchers who repetitively throw at high velocities without scheduling enough rest to allow for adequate ligament repair (Mirowitz and London, 1992). This careful balancing act between catabolic and anabolic processes exists in other tissues. For example, in skeletal muscle and bone remodeling, there is a coordinated process that balances protein degradation vs. protein synthesis and bone resorption vs. bone formation, respectively (McCarthy and Esser, 2010; Raggatt and Partridge, 2010). As for the ACL, evidence of such a “tissue fatigue” injury mechanism comes from *ex vivo* ACL tissue, retrieved from non-contact ACL injured patients during their reconstruction surgery, when compared to ACL tissue from repeatedly-loaded cadaveric knees (Chen et al., 2019; Kim et al., 2022). These cadaveric knees had undergone repeated loading that simulated pivot-landings (i.e., knee compression, flexion, and internal tibial rotation) and were investigated for damage to the ACL. Interestingly, the cadaveric ligaments exhibited disruptions of the collagen in the form of unraveling and voids following repetitive loading (Kim et al., 2022), the same pattern of structural damage that was found in the ACL explants from non-contact ACL injury patients undergoing reconstruction (Chen et al., 2019). In short, results of the present study represent the first *in vivo* evidence that significant increases in sports training dosage, in particular ACL-straining athletic activities and maneuvers, are associated with non-contact ACL injury. This provides indirect support for the “tissue fatigue” injury mechanism for which the ACL does not have enough time to fully repair, thereby leading to the accumulation of microdamage, weakening of the ligament and eventual failure.

Demonstrating that the ACL can fail via a “tissue fatigue” injury mechanism, whereby a rapid increase in the intensity of ACL-straining athletic activities and maneuvers can affect the balance between ACL degradation and recovery, has several implications for injury prevention. For instance, monitoring and limiting athletes’ levels of ACL-straining activities and maneuvers, similar to the use of the pitch count for baseball players in UCL injury prevention, may help prevent some non-contact ACL injuries (Wojtys et al., 2016). Doing so would give the ACL enough time to recover between bouts of large strain and thus ligament homeostasis could be maintained. This does not preclude training activities and maneuvers known to not significantly load the ACL such as running without sharp turns, for example,. For ACL ‘tissue fatigue’ injury prevention efforts to be successful, many factors that are currently poorly characterized need to be investigated and taken into consideration. First, the healing rate of the ACL is poorly understood although we know that it is relatively slow compared to other tissues such as bone, cartilage, and muscle (Panjabi, 2006; Jung et al., 2009; Nyland et al., 2022), thus making the ACL particularly susceptible to the accumulation of microdamage from repetitive submaximal loading cycles. Second, the location of the microdamage within the ligament should also be considered because the ligament insertion sites take longer to remodel than other regions (Arnoczky, 1983; Toy et al., 1995). Third, sex differences in ACL healing rate should be investigated. For example, female athletes experience ACL injuries 2–10 times more frequently than males due to several established physiological and anatomical factors (Sturnick et al., 2015; Shultz et al., 2019; Barnum et al., 2021). Perhaps a sex difference in ligament healing rate is another contributing factor. If such a sex difference exists, it could have affected our results since the majority of the ACL-injured patients were females. Fourth, nutrition and sleep should be considered as well given that these factors can contribute to the integrity of the ACL’s extracellular matrix (Dodt et al., 1997; Kjaer et al., 2009; Nyland et al., 2022). Lastly, age differences exist in the turnover rate of collagen and should be taken into consideration (Sivan et al., 2008). Additionally, with the hamstrings and quadriceps known as agonists and antagonists, respectively, to the ACL, athletes can train to perform their respective sport maneuvers in a way that minimizes ACL strain but without compromising performance (Withrow et al., 2006; 2008; Wojtys et al., 2016). This concept of optimizing maneuvers is important given the known inverse relationship between ACL load and number of loading cycles to ligament failure (Lipps et al., 2013). *In vitro* repetitive loading of human knees revealed that when the magnitude of the repetitive load applied to the knee decreased, the number of loading cycles needed to fail the ACL increased. Therefore, if athletes are able to optimize their maneuvers to consistently keep the strain placed on the ACL below a certain threshold by using a monitoring system, for example, it is possible that many ACL fatigue injuries could be prevented effectively.

The significant decrease in weightlifting and summative non-risky athletic activities and maneuvers in contact ACL-injured patients is an interesting result. Given that the summative non-risky activity score combined the scores of only the weightlifting and running activities, this significant decrease in non-risky activity levels was most likely due to the significant decrease in weightlifting activity level. Accordingly, this decrease in weightlifting activity might have resulted in the patients’ muscle strength not being maintained during the 6-month period leading up to injury. Although most contact injuries are probably a result of a single event that places the healthy ACL under excessively large strain, where the accumulation of microdamage in the ligament plays a minimal role, a decrease in muscle strength may cause the knee to become more susceptible to injury for a variety of reasons. Muscle strength is important for controlling lower limb dynamic stability and aids in resisting muscle fatigue, which is associated with decreased performance (Schipplein and Andriacchi, 1991; Besier et al., 2003; Hunter et al., 2004). In fact, loss of lower extremity muscle strength has been associated with greater risk of sustaining a traumatic knee injury in females (Ryman Augustsson and Ageberg, 2017). Therefore, the reduction in weightlifting activity may have placed the knee and its ACL at an increased risk of injury.

Some limitations of our study should be noted. First, since this was a retrospective survey, it is subject to recall bias. We relied on the patients to accurately recall their activity levels up to 6 months before their ACL injury. It probably helped that the majority (57%) of the patients completed the questionnaire within 1 month of injury, with 85% completing it within 2 months, and 89% within 3 months, thereby limiting the recall period and potentially reducing recall bias. In addition, many athletes have set schedules for their extracurricular activities, which can ease recall. Second, the questionnaire only assessed levels of athletic activities and maneuvers at three time points (6 months, 3 months, and 1 week) prior to injury. This was implemented by design in order to balance the collection of detailed data with the questionnaire’s time burden on the patients and their willingness and ability to complete it adequately. Thus, it did not capture any changes in activities or maneuvers that may have occurred during the 3-month intervals between these time points. Nevertheless, we are confident that this 3-month interval captured any significant changes that would affect ACL integrity given the slow turnover rate of the ACL collagen (Rucklidge et al., 1992). Third, our sample size was modest, especially when assessing non-contact and contact ACL injury groups separately; nevertheless, we found statistically significant changes in activity levels in the 6-month period leading up to ACL injury. Building on our finding that sport-specific drills activity significantly increased in non-contact injury patients, future research should increase the sample size with a focus on non-contact injury patients. This could uncover other athletic activities or maneuvers that exhibit a similar pattern. Fourth, there were missing data for several patients. However, our statistical approach—the linear mixed-effects regression model—allowed for the retention of all data from all patients as opposed to alternate approaches (e.g., analysis of variance), which removes cases with missing data from the analysis. Lastly, we did not account for other contributing factors to injury risk such as the patients’ nutrition, sleep, and use of a knee brace. However, given that the athletes were largely amateurs (school-aged or recreational adult athletes), we suspect that such factors confounded our study’s results minimally, if at all.

Ultimately, the levels of several athletic activities/maneuvers of ACL-injured patients changed in the 6 months leading up to their injury, including a significant increase in levels of sport-specific drills among patients who sustained a non-contact injury and a significant decrease in weightlifting activity among those who sustained a contact injury. These results provide evidence that a rapid increase in levels of activities/maneuvers known to apply large forces on the ACL may be a risk factor for injuries to this ligament, especially for non-contact ACL injuries, which has implications for training and injury prevention. Future research should aim to provide direct *in vivo* evidence of microdamage accumulation in the ACL, for instance by means of serum biomarkers (Svoboda et al., 2016) or magnetic resonance imaging (Barnes et al., 2023).

## Data Availability

The raw data supporting the conclusion of this article will be made available by the authors, without undue reservation.
