# Supplementary material for: Levels of ACL-straining activities increased in the six months prior to non-contact ACL injury in a retrospective survey: evidence consistent with ACL fatigue failure
Source: Front Physiol. 2023 May 5;14:1166980. doi: 10.3389/fphys.2023.1166980 (PMC10198379; doi:10.3389/fphys.2023.1166980)
Supplement: Supplementary file 1 [file DataSheet2.pdf]

Patient Name: Involved Knee: R / L Date of Visit:

Age: Sex: M / F Height (in): Weight (lbs):

Injury Information:

Date of Injury (MM/YYYY):

Injury Diagnosis:

Time-point of occurrence during sport: Pre-season / In-season / Off-season

Injury Mechanism: Non-contact / Contact

Previous Musculoskeletal Injuries:

Sports Activity Information:

Primary Sport(s):

At what age did you begin competitive sports participation?

Please indicate the **TYPE** of activity at the **TIME (1 week prior)** of your injury:

- Weightlifting .... ☐ **Yes** ☐ **No**
- Sport-specific drills .... ☐ **Yes** ☐ **No**
- Running .... ☐ **Yes** ☐ **No**
- Jumping .... ☐ **Yes** ☐ **No**
- Cutting .... ☐ **Yes** ☐ **No**
- Pivoting/Twisting .... ☐ **Yes** ☐ **No**
- Decelerating .... ☐ **Yes** ☐ **No**

Please indicate the **FREQUENCY** of activity at the **TIME (1 week prior)** of your injury:

- Weightlifting .... **minutes/day** = ___________ **days/week** = ___________
- Sport-specific drills .... **minutes/day** = ___________ **days/week** = ___________
- Running .... **minutes/day** = ___________ **days/week** = ___________
- Jumping .... **minutes/day** = ___________ **days/week** = ___________
- Cutting .... **minutes/day** = ___________ **days/week** = ___________
- Pivoting/Twisting .... **minutes/day** = ___________ **days/week** = ___________
- Decelerating .... **minutes/day** = ___________ **days/week** = ___________

Please indicate the **INTENSITY** of activity at the **TIME** of your injury **(0=Not intense; 10=Most intense)**:

- Weightlifting **0 1 2 3 4 5 6 7 8 9 10**
- Sport-specific drills **0 1 2 3 4 5 6 7 8 9 10**
- Running **0 1 2 3 4 5 6 7 8 9 10**
- Jumping **0 1 2 3 4 5 6 7 8 9 10**
- Cutting **0 1 2 3 4 5 6 7 8 9 10**
- Pivoting/Twisting **0 1 2 3 4 5 6 7 8 9 10**
- Decelerating **0 1 2 3 4 5 6 7 8 9 10**

<u>3 Months Prior to Injury</u>

Please indicate the **TYPE** of activity **3 MONTHS PRIOR** to your injury:

- Weightlifting .... ☐ **Yes** ☐ **No**
- Sport-specific drills .... ☐ **Yes** ☐ **No**
- Running .... ☐ **Yes** ☐ **No**
- Jumping .... ☐ **Yes** ☐ **No**
- Cutting .... ☐ **Yes** ☐ **No**
- Pivoting/Twisting .... ☐ **Yes** ☐ **No**
- Decelerating .... ☐ **Yes** ☐ **No**

Please indicate the **FREQUENCY** of activity **3 MONTHS PRIOR** to your injury:

- Weightlifting .... **minutes/day** = ____________ **days/week** = ____________
- Sport-specific drills .... **minutes/day** = ____________ **days/week** = ____________
- Running .... **minutes/day** = ____________ **days/week** = ____________
- Jumping .... **minutes/day** = ____________ **days/week** = ____________
- Cutting .... **minutes/day** = ____________ **days/week** = ____________
- Pivoting/Twisting .... **minutes/day** = ____________ **days/week** = ____________
- Decelerating .... **minutes/day** = ____________ **days/week** = ____________

Please indicate the **INTENSITY** of activity **3 MONTHS PRIOR** to your injury
(0=Not intense; 10=Most intense):

- Weightlifting **0 1 2 3 4 5 6 7 8 9 10**
- Sport-specific drills **0 1 2 3 4 5 6 7 8 9 10**
- Running **0 1 2 3 4 5 6 7 8 9 10**
- Jumping **0 1 2 3 4 5 6 7 8 9 10**
- Cutting **0 1 2 3 4 5 6 7 8 9 10**
- Pivoting/Twisting **0 1 2 3 4 5 6 7 8 9 10**
- Decelerating **0 1 2 3 4 5 6 7 8 9 10**

6 Months Prior to Injury

Please indicate the **TYPE** of activity **6 MONTHS PRIOR** to your injury:

- Weightlifting .... ☐ **Yes** ☐ **No**
- Sport-specific drills .... ☐ **Yes** ☐ **No**
- Running .... ☐ **Yes** ☐ **No**
- Jumping .... ☐ **Yes** ☐ **No**
- Cutting .... ☐ **Yes** ☐ **No**
- Pivoting/Twisting .... ☐ **Yes** ☐ **No**
- Decelerating .... ☐ **Yes** ☐ **No**

Please indicate the **FREQUENCY** of activity **6 MONTHS PRIOR** to your injury:

- Weightlifting .... **minutes/day** = ____________ **days/week** = ____________
- Sport-specific drills .... **minutes/day** = ____________ **days/week** = ____________
- Running .... **minutes/day** = ____________ **days/week** = ____________
- Jumping .... **minutes/day** = ____________ **days/week** = ____________
- Cutting .... **minutes/day** = ____________ **days/week** = ____________
- Pivoting/Twisting .... **minutes/day** = ____________ **days/week** = ____________
- Decelerating .... **minutes/day** = ____________ **days/week** = ____________

Please indicate the **INTENSITY** of activity **6 MONTHS PRIOR** to your injury
(0=Not intense; 10=Most intense):

- Weightlifting **0 1 2 3 4 5 6 7 8 9 10**
- Sport-specific drills **0 1 2 3 4 5 6 7 8 9 10**
- Running **0 1 2 3 4 5 6 7 8 9 10**
- Jumping **0 1 2 3 4 5 6 7 8 9 10**
- Cutting **0 1 2 3 4 5 6 7 8 9 10**
- Pivoting/Twisting **0 1 2 3 4 5 6 7 8 9 10**
- Decelerating **0 1 2 3 4 5 6 7 8 9 10**
