# Supplementary material for: Levels of ACL-straining activities increased in the six months prior to non-contact ACL injury in a retrospective survey: evidence consistent with ACL fatigue failure
Source: Front Physiol. 2023 May 5;14:1166980. doi: 10.3389/fphys.2023.1166980 (PMC10198379; doi:10.3389/fphys.2023.1166980)
Supplement: Supplementary file 2 [file DataSheet1.PDF]

Supplementary Material

Levels of ACL-straining activities increased in the six months leading up to non-contact ACL injury: in vivo evidence for ACL fatigue failure mechanism

Louis H. Grodman, Mélanie L. Beaulieu, James A. Ashton-Miller, Edward M. Wojtys*
***Correspondence:** Edward M. Wojtys, MD: edwojtys@med.umich.edu

SPORT & PHYSICAL ACTIVITY QUESTIONNAIRE

Patient Name: Involved Knee: R / L Date of Visit:

Age: Sex: M / F Height (in): Weight (lbs):

Injury Information:

Date of Injury (MM/YYYY):

Injury Diagnosis:

Time-point of occurrence during sport: Pre-season / In-season / Off-season

Injury Mechanism: Non-contact / Contact

Previous Musculoskeletal Injuries:

Sports Activity Information:

Primary Sport(s):

At what age did you begin competitive sports participation? :

Please indicate the **TYPE** of activity at the **TIME (1 week prior)** of your injury:

- Weightlifting .... ☐ **Yes** ☐ **No**
- Sport-specific drills .... ☐ **Yes** ☐ **No**
- Running .... ☐ **Yes** ☐ **No**
- Jumping .... ☐ **Yes** ☐ **No**
- Cutting .... ☐ **Yes** ☐ **No**
- Pivoting/Twisting .... ☐ **Yes** ☐ **No**
- Decelerating .... ☐ **Yes** ☐ **No**

Please indicate the **FREQUENCY** of activity at the **TIME (1 week prior)** of your injury:

- Weightlifting .... **minutes/day =** ___________ **days/week =** ___________
- Sport-specific drills .... **minutes/day =** ___________ **days/week =** ___________
- Running .... **minutes/day =** ___________ **days/week =** ___________
- Jumping .... **minutes/day =** ___________ **days/week =** ___________
- Cutting .... **minutes/day =** ___________ **days/week =** ___________

- Pivoting/Twisting........... **minutes/day =** ___________ **days/week =** ___________
- Decelerating .... **minutes/day =** ___________ **days/week =** ___________

Please indicate the **INTENSITY** of activity at the **TIME** of your injury **(0=Not intense; 10=Most intense)**:

- Weightlifting **0 1 2 3 4 5 6 7 8 9 10**
- Sport-specific drills **0 1 2 3 4 5 6 7 8 9 10**
- Running......................... **0 1 2 3 4 5 6 7 8 9 10**
- Jumping......................... **0 1 2 3 4 5 6 7 8 9 10**
- Cutting **0 1 2 3 4 5 6 7 8 9 10**
- Pivoting/Twisting........... **0 1 2 3 4 5 6 7 8 9 10**
- Decelerating **0 1 2 3 4 5 6 7 8 9 10**

3 Months Prior to Injury

Please indicate the **TYPE** of activity **3 MONTHS PRIOR** to your injury:

- Weightlifting .... ☐ **Yes** ☐ **No**
- Sport-specific drills .... ☐ **Yes** ☐ **No**
- Running.......................... ☐ **Yes** ☐ **No**
- Jumping.......................... ☐ **Yes** ☐ **No**
- Cutting .... ☐ **Yes** ☐ **No**
- Pivoting/Twisting............ ☐ **Yes** ☐ **No**
- Decelerating .... ☐ **Yes** ☐ **No**

Please indicate the **FREQUENCY** of activity **3 MONTHS PRIOR** to your injury:

- Weightlifting .... **minutes/day =** ___________ **days/week =** ___________
- Sport-specific drills .... **minutes/day =** ___________ **days/week =** ___________
- Running......................... **minutes/day =** ___________ **days/week =** ___________
- Jumping......................... **minutes/day =** ___________ **days/week =** ___________
- Cutting .... **minutes/day =** ___________ **days/week =** ___________
- Pivoting/Twisting........... **minutes/day =** ___________ **days/week =** ___________
- Decelerating .... **minutes/day =** ___________ **days/week =** ___________

Please indicate the **INTENSITY** of activity **3 MONTHS PRIOR** to your injury
(0=Not intense; 10=Most intense):

- Weightlifting **0 1 2 3 4 5 6 7 8 9 10**
- Sport-specific drills **0 1 2 3 4 5 6 7 8 9 10**
- Running......................... **0 1 2 3 4 5 6 7 8 9 10**
- Jumping......................... **0 1 2 3 4 5 6 7 8 9 10**
- Cutting **0 1 2 3 4 5 6 7 8 9 10**
- Pivoting/Twisting........... **0 1 2 3 4 5 6 7 8 9 10**
- Decelerating **0 1 2 3 4 5 6 7 8 9 10**

6 Months Prior to Injury

Please indicate the **TYPE** of activity **6 MONTHS PRIOR** to your injury:

- Weightlifting .... ☐ **Yes** ☐ **No**
- Sport-specific drills .... ☐ **Yes** ☐ **No**
- Running .... ☐ **Yes** ☐ **No**
- Jumping .... ☐ **Yes** ☐ **No**
- Cutting .... ☐ **Yes** ☐ **No**
- Pivoting/Twisting .... ☐ **Yes** ☐ **No**
- Decelerating .... ☐ **Yes** ☐ **No**

Please indicate the **FREQUENCY** of activity **6 MONTHS PRIOR** to your injury:

- Weightlifting .... **minutes/day =** ___________ **days/week =** ___________
- Sport-specific drills .... **minutes/day =** ___________ **days/week =** ___________
- Running .... **minutes/day =** ___________ **days/week =** ___________
- Jumping .... **minutes/day =** ___________ **days/week =** ___________
- Cutting .... **minutes/day =** ___________ **days/week =** ___________
- Pivoting/Twisting .... **minutes/day =** ___________ **days/week =** ___________
- Decelerating .... **minutes/day =** ___________ **days/week =** ___________

Please indicate the **INTENSITY** of activity **6 MONTHS PRIOR** to your injury
(0=Not intense; 10=Most intense):

- Weightlifting **0 1 2 3 4 5 6 7 8 9 10**
- Sport-specific drills **0 1 2 3 4 5 6 7 8 9 10**
- Running **0 1 2 3 4 5 6 7 8 9 10**
- Jumping **0 1 2 3 4 5 6 7 8 9 10**
- Cutting **0 1 2 3 4 5 6 7 8 9 10**
- Pivoting/Twisting **0 1 2 3 4 5 6 7 8 9 10**
- Decelerating **0 1 2 3 4 5 6 7 8 9 10**
